# Scrub Typhus and Comparisons of Four Main Ethnic Communities in Taiwan in 2004 versus 2008 Using Geographically Weighted Regression

**DOI:** 10.5539/gjhs.v5n3p101

**Published:** 2013-02-15

**Authors:** Pui-Jen Tsai

**Affiliations:** 1 The Center for General Education, Aletheia University, New Taipei, Taiwan

**Keywords:** scrub typhus, geographically weighted regression, *kappa* statistic, Taiwanese ethnic communities, disease map

## Abstract

**Purpose:**

On the main island of Taiwan, a higher risk of scrub typhus infection has been reported in endemic clusters in Southeastern Taiwan and in mountainous township areas. However, research on health care problems associated with scrub typhus in Taiwanese ethnic peoples is limited. This study employs spatial analysis of areal data to determine spatial features related to scrub typhus and the four main Taiwanese ethnicities: Hoklo, Hakka, Mainlander, and aboriginal communities, respectively.

**Methods:**

We used a GWR spatial method to analyze the local regressed relationships between scrub typhus incidence and ethnic community percentage in 349 townships in Taiwan, and the subsequent spatial regressed resultants and local parameter estimates were compared between two periods of 2004 and 2008 by *kappa* statistics.

**Results:**

In the GWR models, the spatial regressed relationships of scrub typhus incidences and the Hoklo communities showed significant and negative parameter estimates in numerous locations, showing clusters in Southeastern and Southwestern Taiwan, and areas of the central and southern mountainous townships. Both Hakka and Mainlander communities in the mountainous townships showed less-regressed clusters with scrub typhus prevalence. However, clusters of Aboriginal populations were positively correlated with scrub typhus in highly infected mountainous areas and in Southeastern Taiwan. The kappa value results and the comparisons of local parameter estimates in the 349 townships in Taiwan between 2004 and 2008 indicated that the incidence of scrub typhus in the Hoklo communities was substantial, in the Hakka communities was fair, in the Mainlander communities was slight, and in the aboriginal communities was moderate, respectively.

**Conclusion:**

The aboriginal communities have been closely associated with higher risks of scrub typhus in the mountainous townships and in the southeastern portion of Taiwan.

## 1. Introduction

Scrub typhus is a vector-borne zoonotic disease caused by *Orientia tsutsugamushi*, in which intracellular parasites live within the cells of other animals. *O. tsutsugamushi* lives primarily in mites belonging to the species *Leptotrombidium* (*Trombicula*) *akamushi* and *Leptotrombidium deliense* (Beers & Berkow, 2004). Scrub typhus infects approximately 1 million people annually, and a billion more are estimated to be at risk (Kawamura et al., 1995; Rosenberg, 1997). Because the disease is limited to Eastern and Southeastern Asia, India, Northern Australia, and adjacent islands, it is also commonly referred to as tropical typhus (Beers & Berkow, 2004; Devine, 2003). The infection is transmitted to humans and rodents by various species of infective trombiculid mites that feed on lymph and tissue fluid rather than blood. The mites have a 4-stage life cycle: egg, larva, nymph, and adult. The larval stage is the only stage that transmits the disease to humans and other vertebrates. In regions where scrub typhus is a constant threat, a natural cycle of *O. tsutsugamushi* transmission occurs between mite larvae and small mammals (e.g., field mice and rats). Humans enter the cycle of rickettsial infection only accidentally (Suputtamongkol et al., 2009). The seasonal occurrence of scrub typhus varies with the climate in different countries, and occurs more frequently during the rainy season. Forest clearings, riverbanks, and grassy regions provide optimal conditions for the infected mites to thrive. These small geographic regions are high-risk areas for humans, and have been called scrub-typhus islands (Beers & Berkow, 2004). Scrub typhus occurrence is frequently related to temperature, and occasionally to rainfall (Kawamura et al., 1995; Olson, 1979; Olson & Scheer, 1978; Traub & Wisseman, 1974). The relationship between scrub typhus incidence and climate reflects chigger responses to the environment (Kawamura et al., 1995).

From 2003 to 2008, 1558 confirmed scrub typhus infections were reported on the main island of Taiwan. The mean incidence rate was 1.15 cases per year per 100,000 residents, and it was significantly higher for men than for women, increasing monotonically in the age range of 60-69 years (Kuo et al., 2011a). A higher risk of scrub typhus infection was reported in the Matuo Islands (Lienchiang County), Kinmen (Kinmen County), the Pescadore Islands (Penghu County), in endemic clusters in Southeastern Taiwan (plain townships in Hualien County and Taitung County), and in mountainous township areas on the main island of Taiwan (Lee et al., 2006; Huang et al., 2009; Kuo et al., 2011a; Centers for Disease Control, Taiwan, 2011). Numerous reports have documented that habitation in aboriginal townships is a vital factor closely associated with the incidence and prevalence of scrub typhus in Taiwan (Kuo et al., 2011a; Centers for Disease Control, Taiwan, 2011). However, research on health care problems associated with scrub typhus in Taiwanese ethnic peoples is limited. We thus employ a spatial analysis of the areal data to determine spatial features related to scrub typhus and the four major Taiwanese communities.

## 2. Method

### 2.1 Study Area

**Figure 1:**
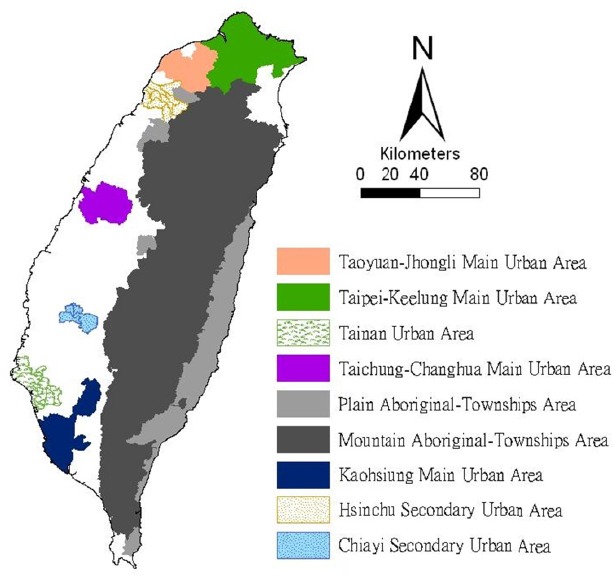
Map of urban areas and aboriginal townships in the study area Map of the study area divided into 349 administrative districts, including seven urban areas and an integrated area of 54 plains and mountainous aboriginal townships.

The study was conducted within the main island of Taiwan (excluding all islets), which, in 2008, comprised more than 23 million inhabitants living in an area of 36,000 km^2^. A total of 349 local administrative government areas, including five main urban areas, two secondary urban areas, 162 rural townships, and 54 plain and mountainous aboriginal townships, were assessed (Figure 1). According to a bulletin issued in 2002 from the Ministry of the Interior, urban areas are regions with at least one metropolitan center and can include neighboring cities and townships that share socioeconomic activities. Main urban areas are defined as those with a population larger than 1 million, specifically Taipei-Keelung, Kaohsiung, Taichung-Changhua, Jhongli-Taoyuan, and Tainan. Secondary urban areas are defined as those with a residential population ranging from 0.3 to 1 million (Hsinchu and Chiayi).

### 2.2 Data Collection and Management

The percentages of the four major Taiwanese ethnic communities in each township were obtained from an official report of the Council for Hakka Affairs (2004 and 2008; Council for Hakka Affairs, 2012). According to self-reports in official governmental statistics, Han Chinese constitute 98% of the Taiwan population, whereas Taiwanese aborigines constitute the remaining 2%. The composite category of the “Taiwanese resident” is often reputed to include a significant population of at least four constituent ethnic groups: the Hoklo (71.3%), the Hakka (13.8%), the Mainlander (8.5%), and the Taiwanese aborigines (1.9%; Council for Hakka Affairs, 2012).

Data for confirmed cases of scrub typhus were obtained from the Notifiable Infectious Diseases Statistics System and Infectious Diseases Database at the Taiwanese Center for Disease Control (Department of Health, 2012). The Ministry of the Interior provided the demographic data used in this study (Ministry of the Interior, 2012). The age-adjusted standard incidence rates were calculated with a direct adjustment using the global population in 2000 as the standard population (Ahmad et al., 2001). The age-adjusted standard incidence rates from 2000–2010 were calculated. The standardized incidence ratio (SIR) of scrub typhus was calculated for each township and then used as the response variable in the GWR model. The GWR model used the following explanatory variables: percentage of the Hoklo, Hakka, Mainlander, and aboriginal communities.

### 2.3 Geographically Weighted Regression

The GWR model extends the traditional standard regression framework that estimates local, rather than global, parameters (Fotheringham et al., 1998). The model is a type of local statistic that produces a set of local parameter estimates showing how a relationship varies over space. This enables examining the spatial pattern of the local estimates to gain a better understanding of possible hidden causes for this pattern (Fotheringham et al., 2002). Conversely, a traditional regression method, such as ordinary least squares (OLS), is a type of global statistic that assumes that the relationship under study is constant over space, and therefore, assumes that the parameter is the same for the entire study area.

An OLS model can be defined as follows:


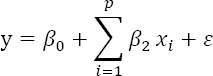


where y is the response variable, β_0_ is the intercept, β_i_ is the parameter estimate (coefficient) for the explanatory variable x_i_, p is the number of explanatory variables, and ε is the error term.

The GWR model allows local, rather than global, parameters to be estimated for the study area. Thus, the GWR model rewrites the OLS model as follows:


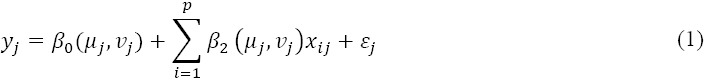


where u_j_ and v_j_ are the coordinates for each location j, β_0_ (u_j_,v_j_) is the intercept for location j, and β_i_ (u_j_,v_j_) is the local parameter estimate for the explanatory variable x_i_ at location j.

The weight assigned to each observation is based on a distance-decay function centered on observation i.

The estimator for the GWR model is similar to the weighted least squares (WLS) global model, except that the weights are conditioned on the location u relative to the observations in the data set, and hence, they change for each location. The estimator takes the following form:


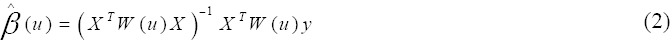


W (u) is the square matrix of weights relative to the position u. A particular location can be indexed (u_j_, v_j_) in the study area. X^T^W (u)X is the geographically weighted variance-covariance matrix, and y is the vector of the value of the response variable.

The W (u) matrix contains the geographical weights in its leading diagonal, and zero in its off-diagonal elements.


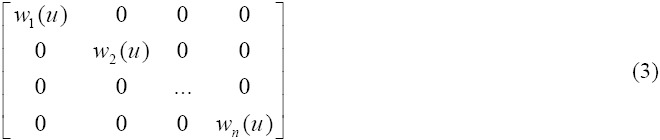


In the area in which this study was conducted, the sample points produced by the polygon centroids were not regularly placed, but were clustered. A convenient way to implement the adaptive bandwidth specification is to select a kernel that allows the same number of sample points for estimations. The weight can then be calculated using the specified kernel and the value set for any observation with a distance that exceeds the bandwidth to zero. The bi-square function is as follows:


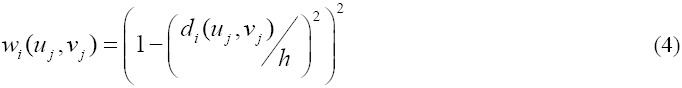


where wi (uj,vj) is zero when di (vj,uj) > h. The term h represents a quantity known as the bandwidth. This is a near-Gaussian function with the useful property of the weight being zero at a finite distance.

The bandwidth was chosen by minimizing the Akaike information criterion (AIC) score, calculated as follows:


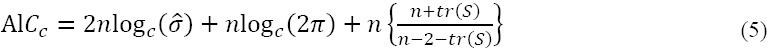


where tr(S) is the trace of the hat matrix. The AIC method has the advantage of accounting for the fact that the degrees of freedom may vary among models centered on different observations. The optimal bandwidth was determined by minimizing the corrected AIC, as described by Fotheringham et al. (2002). The GWR models produce a set of local regression results, including local parameter estimates and local residuals, which can be mapped to show their spatial variability. Geographically weighted regressions were employed and mapped using ArcMap 9.3.

We used the Benjamini-Hochberg (B-H) procedure to control the false discovery rate, which consistently modifies the significance level for each test. This procedure was used to determine the significance of parameter estimates produced by the GWR model. Thissen et al. (2002) proposed a quick and easy method for calculating the B-H procedure false discovery rate using Microsoft Excel (Thissen et al., 2002). The B-H approach controls the FDR by sequentially comparing the observed *p* value for each of a family of multiple test statistics (from largest to smallest) to a list of computed B-H critical values [*pB-H*(*i*)]. The critical value on the list is determined for each test statistic, and indexed by *i* by linear interpolation between α/2 (for the largest observed *p* value) to (α/2)/*m*, where *m* is the family size (for the smallest of the *P* values). Because the last value is the Bonferroni critical value, the reason for the power gain of B-H relative to the Bonferroni approach is clear; the B-H approach compares only the smallest of the *m* observed *p* values to the Bonferroni critical value. All other *p* values are calculated using less stringent criteria. The local parameter is estimated to be significant if the *p* value is less than the B-H critical value; otherwise, it is deemed non-significant (Thissen et al., 2002). The results were also used in detecting the spatial similarity between the 2004 and 2008 periods.

### 2.4 Detecting Spatial-Pattern Consistency

The *Kappa* statistic for map comparisons have been developed that provide parametric tests for the similarity of spatial patterns across pairs of variables (Monserud & Leemans, 1992; Hagen, 2003). We used the *Kappa* statistic (Fleiss, 1981), which reflects the consistency between two clustering calculations (i.e., the significant determinations in local parameter estimates across the 2004 and 2008 periods). A value close to 1 represents nearly perfect agreement, whereas values close to or below 0 represent poor agreement. Landis and Koch (1977) developed a useful scale for interpreting the *Kappa* estimate: 0.81–1.00 (*almost perfect*); 0.61–0.80 (*substantial*); 0.41–0.60 (*moderate*); 0.21–0.40 (*fair*); 0.00–0.20 (*slight*); and < 0.0 (*poor*) agreement.

## 3. Results

Table 1 presents a summary of the age-adjusted incidence rates between 2000 and 2010 on the main island of Taiwan, showing that all incidence rates related to men were higher than those for women. Gender ratios, defined as the ratio of men to women, generally ranged from 1 to 2, but increased to 2.29 in 2008.

**Table 1:** Age-adjusted incidence rates of scrub typhus by gender on the main island of Taiwan, from 2000-2010

	Year										
Gender	2000	2001	2002	2003	2004	2005	2006	2007	2008	2009	2010
male*	1.05	1.14	1.03	1.10	1.13	1.55	1.18	1.72	1.70	1.25	1.35
female*	0.61	0.61	0.64	0.71	0.84	1.11	0.81	0.88	0.74	0.66	0.85
male/female ratio	1.72	1.88	1.60	1.54	1.35	1.39	1.46	1.96	2.29	1.90	1.59

* indicates incidence rates per 100,000 people

**Figure 2:**
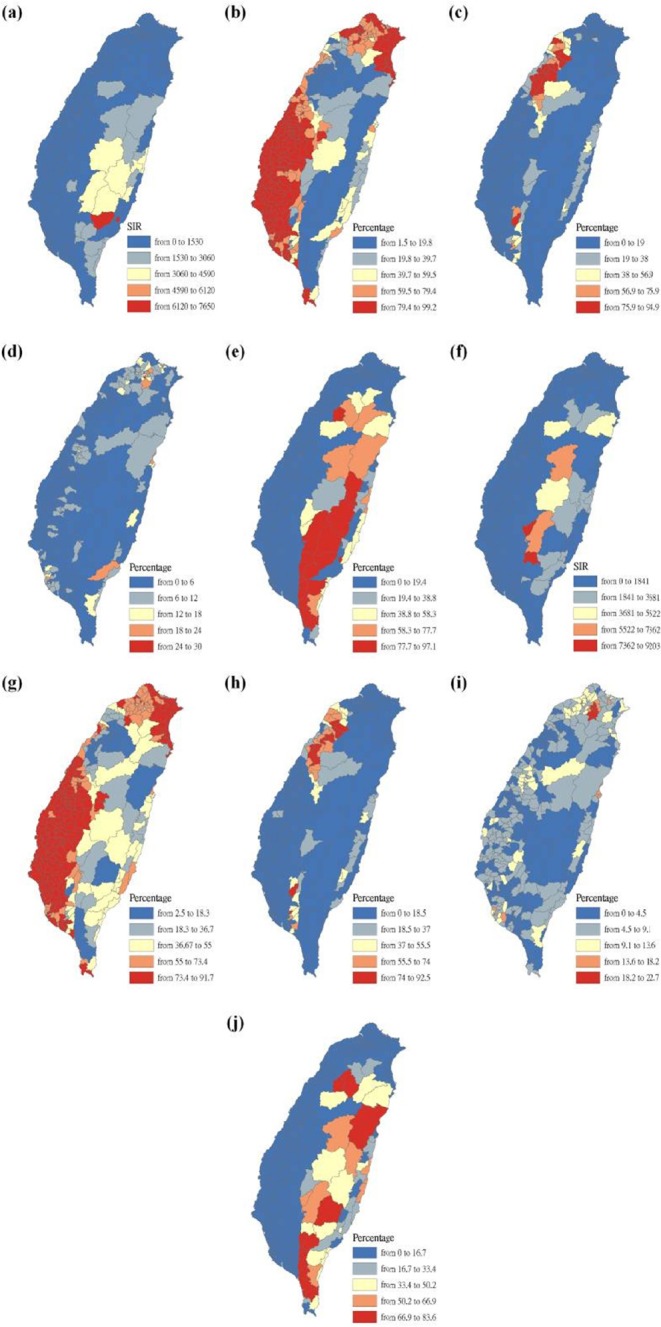
Maps of 349 townships in Taiwan for scrub typhus incidences and four main ethnic communities in 2004 and 2008 Figure (a) shows the standardized incidence ratio (SIR) of scrub typhus in 2004. Figure (b) shows the percentage of Hoklo communities in 2004. Figure (c) shows the percentage of Hakka communities in 2004. Figure (d) shows the percentage of Mainlander communities in 2004. Figure (e) shows the percentage of aboriginal communities in 2004. Figure (f) shows the standardized incidence ratio (SIR) of scrub typhus in 2008. Figure (g) shows the percentage of Hoklo communities in 2008. Figure (h) shows the percentage of Hakka communities in 2008. Figure (i) shows the percentage of Mainlander communities in 2008. Figure (j) shows the percentage of aboriginal communities in 2008.

Figure 2 shows a map of the geographical distributions of the scrub typhus SIR-district and the percentage of the four main Taiwanese ethnicities: the Hoklo, Hakka, Mainlander, and aboriginal communities in Taiwan in 2004 and 2008.

**Figure 3:**
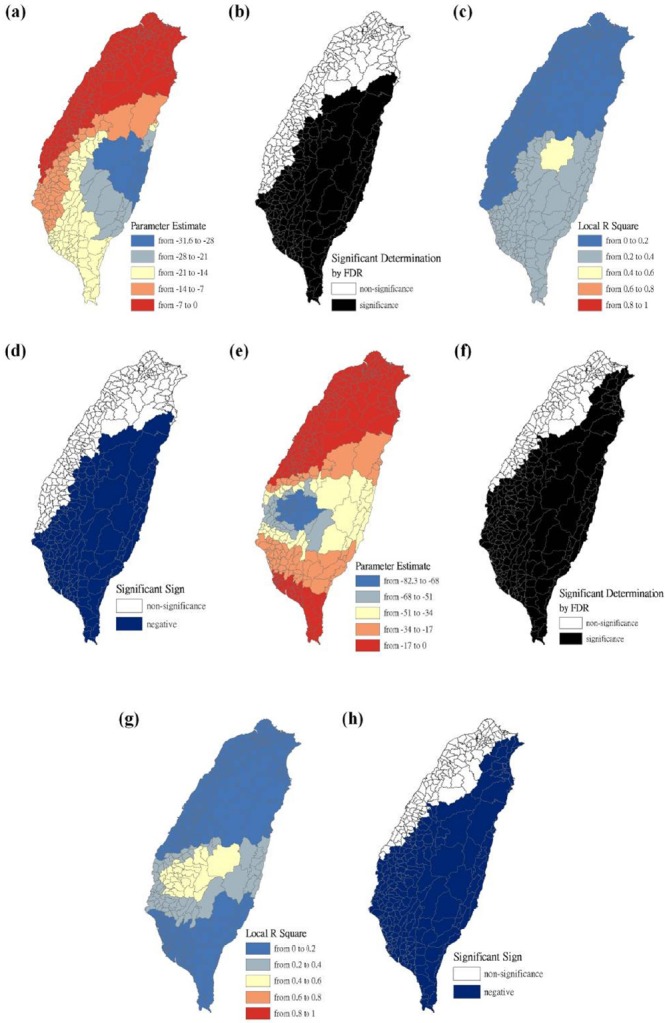
Results of the GWR model for the standardized incidence ratio (SIR) of scrub typhus and the Hoklo communities on the main island of Taiwan in 2004 and 2008 Figure (a) shows the parameter estimate in 2004. Figure (b) shows the significant determination by the false discovery rate (FDR) in 2004. Figure (c) shows the local R^2^ value in 2004. Figure (d) shows the significant sign in 2004. Figure (e) shows the parameter estimate in 2008. Figure (f) shows the significant determination by the false discovery rate (FDR) in 2008. Figure (g) shows the local R^2^ value in 2008. Figure (h) shows the significant sign in 2008.

**Figure 4:**
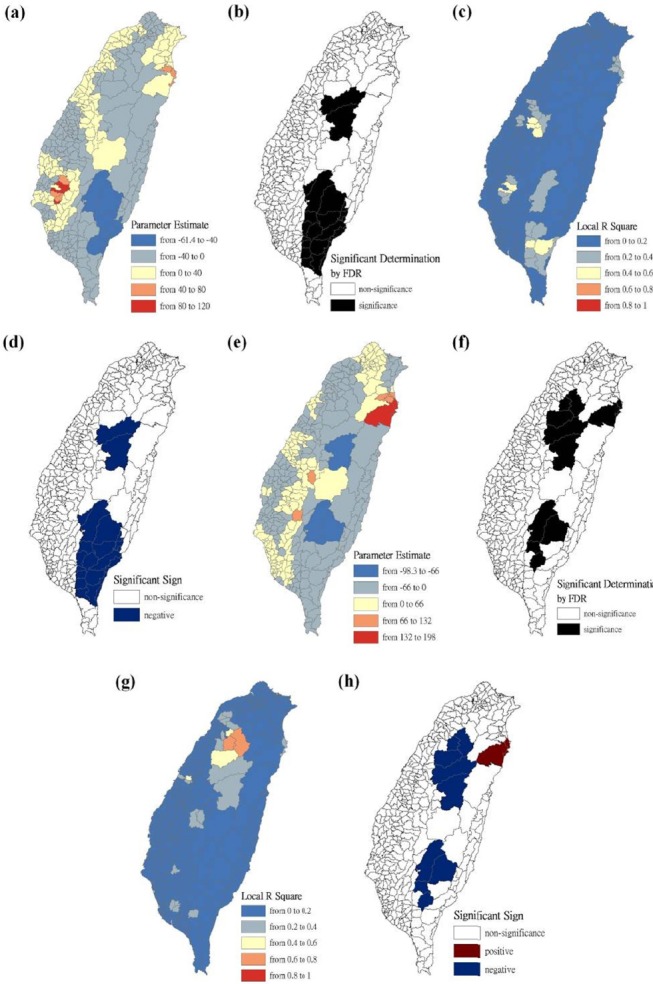
Results of the GWR model for the standardized incidence ratio (SIR) of scrub typhus and the Hakka communities on the main island of Taiwan in 2004 and 2008 Figure (a) shows the parameter estimate in 2004. Figure (b) shows the significant determination by the false discovery rate (FDR) in 2004. Figure (c) shows the local R^2^ value in 2004. Figure (d) shows the significant sign in 2004. Figure (e) shows the parameter estimate in 2008. Figure (f) shows the significant determination by the false discovery rate (FDR) in 2008. Figure (g) shows the local R^2^ value in 2008. Figure (h) shows the significant sign in 2008.

**Figure 5:**
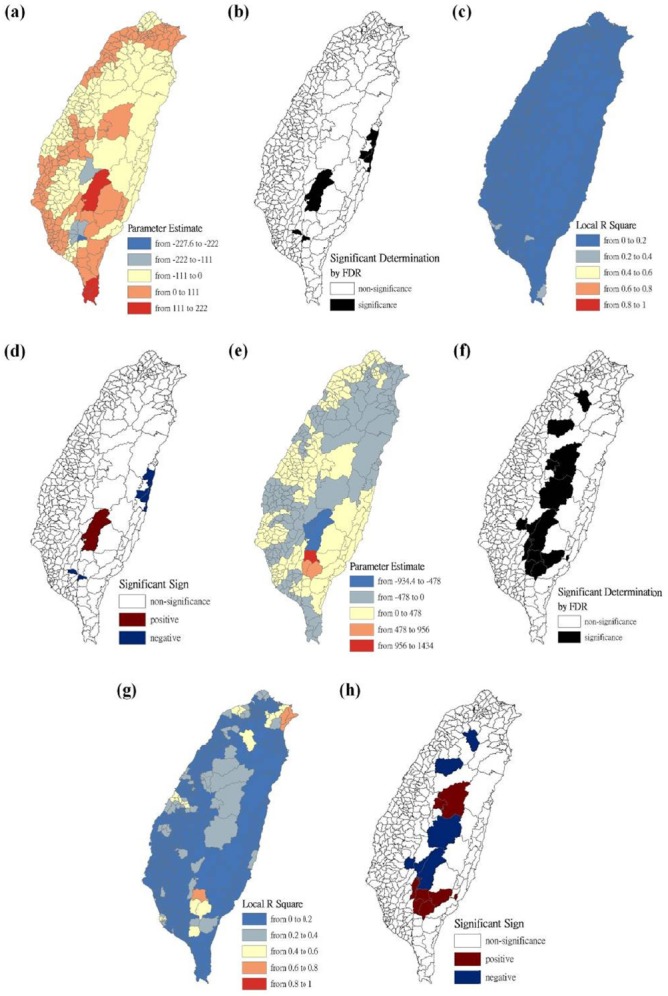
Results of the GWR model for the standardized incidence ratio (SIR) of scrub typhus and the Mainlander communities on the main island of Taiwan in 2004 and 2008 Figure (a) shows the parameter estimate in 2004. Figure (b) shows the significant determination by the false discovery rate (FDR) in 2004. Figure (c) shows the local R^2^ value in 2004. Figure (d) shows the significant sign in 2004. Figure (e) shows the parameter estimate in 2008. Figure (f) shows the significant determination by the false discovery rate (FDR) in 2008. Figure (g) shows the local R^2^ value in 2008. Figure (h) shows the significant sign in 2008

**Figure 6:**
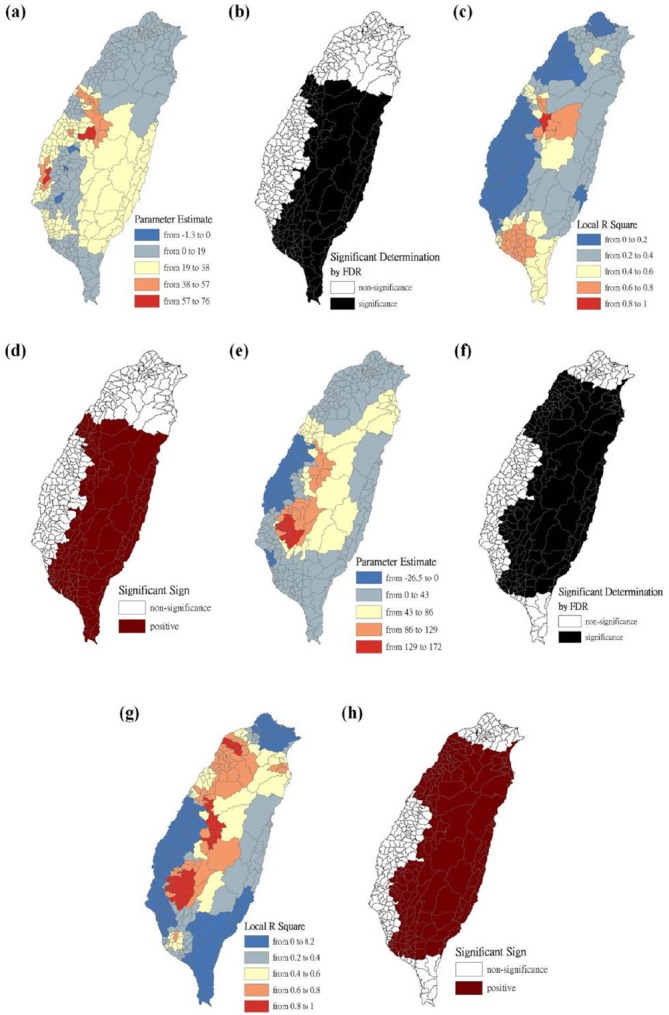
Results of the GWR model for the standardized incidence ratio (SIR) of scrub typhus and the aboriginal communities on the main island of Taiwan in 2004 and 2008 Figure (a) shows the parameter estimate in 2004. Figure (b) shows the significant determination by the false discovery rate (FDR) in 2004. Figure (c) shows the local R^2^ value in 2004. Figure (d) shows the significant sign in 2004. Figure (e) shows the parameter estimate in 2008. Figure (f) shows the significant determination by the false discovery rate (FDR) in 2008. Figure (g) shows the local R^2^ value in 2008. Figure (h) shows the significant sign in 2008.

The maps are presented as parameter estimates, the significant determination of the false discovery rate, local R^2^, and significant sign, in which scrub typhus figures fit the GWR models with explanatory variables of the Hoklo, the Hakka, the Mainlander, and the Aboriginal communities, respectively. In the GWR models, the explanatory variables for the Hoklo communities only showed significant and negative signs of parameter estimates in clusters of most mountainous townships, and in Southwestern and Eastern Taiwan in 2004 and 2008, as shown in Figure 3. The explanatory variables for the Hakka communities showed significant and negative signs of parameter estimates in clusters located in parts of the mountainous township region in both 2004 and 2008. The Suao Township and Nanao Township locations in Yilan County also showed significant and positive signs of parameter estimates only in 2008, as shown in Figure 4. The explanatory variables of Mainlander communities in 2004 showed significant and negative signs of parameter estimates in five townships (i.e., Hualien County of Shoufong Township, Fongbin Township, Guangfu Township, Pingtung County of Yanpu Township, and Majia Township) and had significant and positive signs of parameter estimates in Taoyuan Township in Kaohsiung County. In 2008, seven townships showed significant and positive signs of parameter estimates (i.e., Nantou County of Puli Township. Renai Township, Kaohsiung County of Liouguei Township, Maolin Township, Pingtung County of Sandimen Township, Wutai Township, and Taitung County of Yanping Township), and locations in six townships (i.e., Taoyuan County of Fusing Township, Miaoli County of Taian Township, Nantou County of Sinyi Township, Chiayi County of Dapu Township, Kaohsiung County of Taoyuan Township, and Namasia Township) showed significant and negative signs of parameter estimates, as shown in Figure 5. Figure 6 shows that aboriginal communities were positively and closely related to locations where clusters were covered in most mountainous townships, parts of Central Western and Southwestern Taiwan, and Southeastern Taiwan in 2004 and 2008.

According to the results of local parameter estimates with significant determination, Table 2 shows the results calculated by the *Kappa* statistic, which was used to determine the extent of spatial consistency presented between the two outcomes (i.e., local parameter estimates tested by the false discovery rate in 2004 and 2008) calculated from GWR. The results indicated that the Hoklo communities were substantial and that the *kappa* value was 0.67, the Hakka communities were fair and that *kappa* value was 0.35, the Mainlander communities were slight and that the *kappa* value was 0.08, and the aborigines were moderate and that the *kappa* value was 0.49, respectively.

**Table 2:** Spatial consistency of local parameter estimates calculated by the GWR models, according to comparisons between 2004 and 2008 for ethnic communities in Taiwan

Response variable	Explanatory variable	*Kappa* value	Description
scrub typhus	Hoklo	0.67	substantial
scrub typhus	Hakka	0.35	fair
scrub typhus	Mainlander	0.08	slight
scrub typhus	aboriginals	0.49	moderate

Clusters were calculated by the regression models, in which parameter estimates were significant and positive. Therefore, a higher SIR-district scrub typhus is positively associated with the explanatory variables (i.e., Hakka communities in 2008, Mainlander communities in 2004 and 2008, and aboriginal communities in 2004 and 2008). For cluster-consistency comparisons between 2004 and 2008, the *Kappa* estimate in aboriginal communities was interpreted as moderate and showed a higher consistency than those of Hakka and Mainlander communities. The results indicated that regressed-outcome comparisons between 2004 and 2008 were more similar in aborigines in a 5-year period, but less similar in Hakka and Mainlander communities.

## 4. Discussion

In Taiwan, scrub typhus is the most common rickettsial and notifiable disease, and public health authorities are concerned about its increased incidence (Department of Health, 2012). A higher risk of scrub typhus infection is not only endemic to Southeastern Taiwan and mountainous township area, but also in the Pescadore Islands, Kinmen Islands, and Matou Islands (Centers for Disease Control, 2011). The GWR method is a specific type of spatial regression that generates parameters disaggregated by the spatial units of analysis. We considered analyzing the contiguity-based spatial units (e.g., 349 administrative government areas on the main island of Taiwan) using the GWR method. However, the method was unsuitable for examining isolated regions (e.g., Pescadores, Kinmen, & Matou islands). Therefore, the main island of Taiwan was considered as this study area.

The geographical profile for *O. tsutsugamushi* Hyashi density shows that seropositive outcomes have been observed in captured small rodents and their loaded chigger mites throughout the main island of Taiwan. The main reservoir hosts include *Apodemus agrarius*, *Bandicota indica*, and *Rattus losea*, and the key vector chigger mite is *Leptotrombidium imphalum* (Kuo et al., 2011a, 2011b). *O. tsutsugamushi* was clustered in less developed areas with a relatively low population density, namely the mountainous township area and Southeastern Taiwan; in these areas, a higher incidence of scrub typhus was reported (Kuo et al., 2011a; Kuo et al., 2011b). Frequent human visitation in an endemic area is a critical factor that is increasing the probability of scrub typhus infection, and such visits might also provide more food resources for small rodents. Such factors can enhance the prevalence rate of scrub typhus, such as farm work (Ogawa et al., 2002; Lee et al., 2006; Liu et al., 2009; Kuo et al., 2011a), and troop activity (Payne et al., 2009; Department of Health, 2012). In this study, the habitations and mountainous activities in aboriginal communities provide more chances to contact with the vector chigger mite, leading to much higher infected events than the other ethnic groups. Therefore, the aboriginal communities were closely associated with the prevalence of scrub typhus in the main island of Taiwan. This information can improve planning for the most advantageous types of health care policies and implementing effective health care services.

## 5. Conclusion

The combined method of GWR and kappa statistics is useful for directly analyzing the local regressed relationships between two variables and to detect the two resultant patterns between 2004 and 2008, in which GWR calculated the local parameter estimates of 349 townships on the main island in Taiwan and kappa statistics determined the extent of spatial consistency. Our conclusion indicates that clusters of the aboriginal population have positive significance and correlation to higher risks of scrub typhus incidences in endemic areas of the mountains and in Southeastern Taiwan.
